# Smart Approaches to Food Waste Final Disposal

**DOI:** 10.3390/ijerph16162860

**Published:** 2019-08-10

**Authors:** Franco Cecchi, Cristina Cavinato

**Affiliations:** 1 Department of Biotechnology, University of Verona, Strada le Grazie 15, 37134 Verona, Italy; 2 Department of Environmental Sciences, Informatics and Statistics, University Ca’ Foscari of Venice, Campus Scientifico via Torino 155, 30172 Mestre, Venice, Italy

**Keywords:** anaerobic digestion, codigestion, food waste, organic waste, energy and resource recovery, food waste disposal

## Abstract

Food waste, among the organic wastes, is one of the most promising substrates to be used as a renewable resource. Wide availability of food waste and the high greenhouse gas impacts derived from its inappropriate disposal, boost research through food waste valorization. Several innovative technologies are applied nowadays, mainly focused on bioenergy and bioresource recovery, within a circular economy approach. Nevertheless, food waste treatment should be evaluated in terms of sustainability and considering the availability of an optimized separate collection and a suitable treatment facility. Anaerobic codigestion of waste-activated sludge with food waste is a way to fully utilize available anaerobic digestion plants, increasing biogas production, energy, and nutrient recovery and reducing greenhouse gas (GHG) emissions. Codigestion implementation in Europe is explored and discussed in this paper, taking into account different food waste collection approaches in relation to anaerobic digestion treatment and confirming the sustainability of the anaerobic process based on case studies. Household food waste disposal implementation is also analyzed, and the results show that such a waste management system is able to reduce GHG emissions due to transport reduction and increase wastewater treatment performance.

## 1. Introduction

Food waste anaerobic biological treatment is becoming an important issue within renewable energy recovery systems, even considering the circular economy approach developed in recent years. Three important keynote speeches about the great importance of the anaerobic codigestion process (AcoD), especially sludge and food waste cotreatment, were performed in 2014, where three world conferences related to anaerobic digestion (AD) took place: the 13th Anaerobic Digestion World Congress (AD13, Santiago de Compostela, Spain), the 11th Latin America Anaerobic Digestion Congress (DAAL XI, L’Avana, Cuba), and the 2nd International Conference on Sustainable Solid Waste Management (Athens, Greece). During the AD13 Conference, Prof. Juan Mata-Alvarez and co-authors presented a review of achievements and perspectives about anaerobic digestion and codigestion (AcoD) [1]. The authors observed that the anaerobic codigestion topic was the most relevant within anaerobic digestion research, and in fact 50% of the overall papers published between 2010 and 2013 were about codigestion: the most frequent substrates used were animal manure (54%), sewage sludge (22%), and the organic fraction of municipal solid waste (11%). Considering the type of substrates used, different sectors are involved: agricultural, public services, and industrial. The integration of these sectors requires an increased sharing of competencies, structures, and needs between them. Typically, anaerobic codigestion has been implemented to improve digester yields in terms of energy production from renewable sources, but it is clear that process stability improves with respect to AD of one single type of substrate thanks to nutrient balance. Nevertheless, Mata-Alvarez et al. observed that the full-scale application of AcoD of sewage sludge (SS) with other substrates has not been reported as would be expected, despite the successful integration of SS and the organic fraction of municipal solid waste (OFMSW) reported by Cecchi et al. [2]. Agricultural waste AD had not yet been considered: in fact, it was discussed for the first time at the IV International Symposium on Anaerobic Digestion (ISAD) of solid waste held in Copenhagen (2005) and was successively included in the topic of the 5^th^ ISAD conference on solid waste and energy crops held in Hammamet (2008). According to this scenario, in the keynote speech held during the 2nd International Conference on Sustainable Solid Waste Management (Athens 2014), the role of anaerobic digestion of food waste was presented as a territorial and environmental process [3]. The role of anaerobic digestion of food wastes considering two proposed strategies based on the codigestion approach was discussed: one was anaerobic digestion applied as a service for the agricultural and farming sector, and the other was as a service for citizens (food waste, diapers, and wastewater treatment integration). The union of these two strategies was an environmentally and territorially friendly process that aimed to produce renewable energy and fertilizer material with low greenhouse gas emissions and nutrient recovery.

The 11th Latin America Anaerobic Digestion Congress was an opportunity for Prof. Polanco [4] to talk about the AcoD of food waste together with SS, in an integrated treatment approach implementing a household food waste disposal (FWD). A FWD is an electric device placed under the sink that is used to directly grind food waste into the sewer system together with wastewater and transport it directly to a wastewater treatment plant (WWTP). It was invented in 1927 and was mostly installed in the USA (late 50s) as a hygienic way to dispose of domestic organic wet wastes. In 2008, the FWD installation in USA households was 60% and in Canada 10%, whereas in Australia and New Zealand it was 12% and 30%, respectively [5]: in all of these countries, there were no legislative limits to FWD installation, but local institutions could avoid their use by considering the loading capacity of WWTPs and sewer conditions. In Europe, FWDs’ potential as a waste management strategy has not been fully considered. FWD diffusion has mainly been constrained by controversial opinions and policies that have avoided the direct discharge of food waste into the sewer system. The legislation is made by each member state, so local legislation is adapted to several local aspects, such as the capacity of WWTPs, marketing opportunities for sludge and biogas, cultural attitudes, etc. In the United Kingdom, the installation rate is 5%, the highest among member states, while in other states, the application is still banned (except for Sweden, but only if the unit is connected to a tank) [6]. Recently, Perez et al. [4] discussed and tested the advantages of FWD implementation that gives higher biogas production in an AcoD plant and suggested the feasibility of this approach in terms of energy recovery: by implementing FWDs in half of households, full energy self-sufficiency was reached in the WWTP.

In a study carried out by Iacovidou et al. [7] on the situation of WWTPs and biogas production in the United States, the importance of proper integration of AcoD processes as a way to recover energy and even reduce greenhouse gas (GHG) impacts was highlighted: in fact, the authors reported that wastewater treatment was the eighth largest cause of anthropogenic sources of CH_4_ emissions (in 2012, 12.8 million metric tons of CO_2_ equivalent were produced). Among 14,780 WWTPs, only 1485 digested the sludge produced, but less than 10% of these plants recovered energy (heat and/or electricity) from biogas utilization. The USA is interested in implementing AcoD to increase the efficiency of biogas production and implement combustion or gas-upgrading systems in order to recover heat, electricity, and fuel. Full-scale implementation of food waste and water treatment was observed even in Germany: Krupp et al. [8] investigated the feasibility of codigestion of sewage sludge and food waste in two wastewater treatment plants from both technical and ecological points of view, and they concluded that the codigestion system was advantageous if compared to composting but that food waste must be pretreated properly and hydraulic retention times should be correctly applied.

Recently, Nghiem and colleagues [9] reviewed bottlenecks and possibilities of full-scale codigestion plants treating wastewater sludge and food waste, and they observed that two of the most important issues are the quality of collected food waste and food waste pretreatment technologies. This aspect was confirmed even in Tyagi et al. [10], a paper in which they reviewed the prospective benefits and challenges of food waste anaerobic digestion, highlighting the importance of pretreatment.

Considering the pioneering works of AcoD in Europe [2,11] on integrated food waste and wastewater treatment and the increasing codigestion studies and applications [9], it is possible to consider this approach a promising option both from a process and technological point of view. In order to sustain the advantage of this integration, two successful AcoD (sewage sludge and food waste) processes implemented on a full scale in Italy are discussed below, together with a discussion on the feasibility of using food waste disposals as alternatives to truck collection. The experimental data were complete with some specific details on industrial applications.

## 2. Concept of the Smart Wastewater Treatment Plant with Simultaneous Treatment of Wastewater and Food Waste

A smart wastewater treatment plant must represent a service for a city and territory, where citizens and the environment are the beneficiaries. Within this vision, a WWTP treats both wastewater and the organic fraction of municipal solid waste (OFMSW) obtained within separate collections system. The disposal of the OFMSW in the WWTP could be carried out through different ways: using trucks or through the sewer system after the use of an under-the-sink food waste disposal. Bernstad et al. [12] investigated from a Life Cycle Assessment (LCA) point of view the impact of different collection systems such as paper bags, under-the-sink grinders, vacuum systems, etc., and in all cases, they considered anaerobic digestion and energy and nutrient recovery. They observed that the direct discharge of food waste into the sewer decreased the loss of nutrients and materials that can cause eutrophication, and the codigestion balanced the negative impact with energy recovery and low carbon losses.

**Figure 1:**
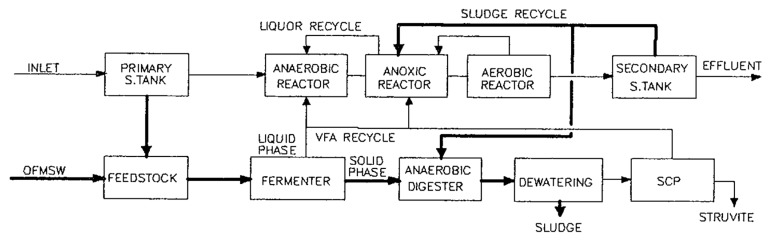
Integrated wastewater and waste treatment scheme [11].

Regardless of the transfer modality, even if it could obviously change the treatment plant’s configuration, what is of interest is the concept that a wet material such as food waste, which contains about 90% water, is treated inside a WWTP suitable for receiving and valorizing it. In fact, thinking about other disposal techniques, an incineration plant is not suitable for the treatment of organic waste because of its limited calorific value: even direct treatment in composting is not optimal, considering the high moisture content of the waste, which requires a substantial amount of structuring material to increase pile porosity. In a smart WWTP, wastewater is treated in a process with Biological Nutrient Removal (nitrogen and phosphorus, BNR) as well as organic carbon. The food waste that comes in the same plant, thanks to its high biodegradability can (after a fermentation step) ensure the effective biological removal of nitrogen and phosphorus, increasing the carbon to nitrogen ratio of the incoming waste. This process scheme was proposed in 1994 (see Figure 1) and realized on a full scale in 1999 (Treviso WWTP): the results of a long-time exercise will be illustrated later on.

In this context, it is possible to increase depurative efficiency (when needed) by extracting liquid fermentation products from biowaste and using them to enhance biological nutrient removal. This strategy avoids the purchase of expensive external carbon sources and complies with discharge limits in sensitive areas (defined by environmental legislation). The solid fraction of OFMSW obtained after the fermentation and separation steps is mixed with sewage sludge produced during the BNR phase and sent to the anaerobic digestion process. This is the codigestion approach of two different matrixes: the result is increased biogas production if compared to sludge alone and a consequent warranty of energy production in facing the thermal energy requirements of a digester. Koch et al. [13] observed that the codigestion of food waste holds promises not only due to a higher methane yield, but in particular due to the accelerated methane production rate, especially when the mixture of raw sludge with food waste is up to a volatile solids ratio of 35%. Moreover, the biomass mixed culture is modified and allows for a higher loading of waste, with a consequently higher yield. The thermal range adopted could be mesophilic (optimum at 35 °C) or thermophilic (optimum at 55 °C): the thermophilic range has some advantages from a thermodynamic point of view, as it in fact has a higher conversion efficiency of complex molecules and a high pathogen removal potential. The solid part of digestate obtained after thermophilic anaerobic digestion treatment is suitable as an agriculture amendment, closing the environmental cycle. The liquid fraction of digestate, rich in nitrogen and phosphorus, remains inside the WWTP and is treated in a crystallization process to obtain struvite ((NH_4_)MgPO_4_·6(H_2_O)), a slow release fertilizer: phosphorus is not a renewable resource, so its recovery is an environmental necessity.

This integrated approach could be considered smart because it is able to guarantee the best treatment results: electrical energy production from a renewable source that can be used directly by the WWTP, agronomic valorization of food waste and sewage sludge, and finally, phosphorus recovery. The unsuccessful applications of AD with organic waste are mainly linked to the quality of the waste, which means the presence of inert material such as plastics, iron, etc. There is a strong necessity to adopt an efficient separate collection system. A door-to-door collection system gives characteristics of the best quality in terms of inert material content and meets the quality requested for the AD process [14]: it is possible to assure the high quality of food waste also by simply implementing an under-the-sink food waste disposal connected to a sewer system.

The smart approach development could even be called a “biorefinery approach”: the cotreatment of different organic wastes through biological technologies could lead to the production of biobased and biodegradable polyesters, such as polyhydroxyalkanoates (PHAs), by using mixed cultures and wastes. This technology is still being studied on a pilot scale because the volumetric productivity is still lower than industrial processes based on pure substrate and microbial cultures. Reis et al. [15] showed that this technology has significant yield, but some technical/scientific aspects need to be overcome in order to upscale the approach in a sustainable way. Some more specific details about food wastes and sewage sludge used as feedstock for an urban biorefinery with the aim of producing biofuels and added-value bioproducts were reported by Battista et al. [16].

Other interesting examples of the “biorefinery approach” are ongoing studies [17] on the anaerobic digestion of separate collected diapers, from which it is possible to recover a volatile fatty acid (VFA) stream usable for PHA production. Other authors have shown that it is possible to accumulate PHAs in sequencing batches (SBR), treating anaerobic digestion supernatants and recovering VFAs from alkaline fermentation with simultaneous nutrient removal [18,19]. The recovery of VFAs has been of great interest recently thanks to their value as chemical building blocks, and food waste fermentation is the most productive substrate giving the best yields [20].

## 3. Material and Methods

### 3.1. Integrated Waste and Wastewater Plant Description

Two cases studies are described below: they represent the first Italian examples of this smart WWTP approach to development. The first project was realized in Treviso, followed by Camposampiero (Padua, PD) and Rovereto (Trento, TN). The first and last WWTPs were monitored by the authors, and then a deeper presentation and discussion was carried out for these two plants.

#### 3.1.1. The Treviso Full-Scale Food Waste Pretreatment and Anaerobic Codigestion Plant

The Treviso WWTP has been operative since 1999: it has 70,000 people equivalents (PEs) of wastewater treatment capacity with a BNR treatment process. The sludge produced is thickened at 3% of total solids and is cotreated with the OFMSW (up to 10 ton day^−1^) in a 2200-m^3^ anaerobic digester. The organic fraction from separate collection was pretreated (within the period considered in this paper) with a low-power system (Patent RN 2004A000038, 2004): the waste was first shredded and sieved with a rotary drum after the first removal of ferromagnetic materials, while those with a low magnetic permeability were removed in a second stage. The substrate thus obtained was reduced in size in a shredder with knives with a span of 15 mm. The biomass was then sent to a wet separator (mixer/separator), where the total solid content was lowered to 7%–8% using the sewage sludge coming from the wastewater treatment line. Here, light and heavy wastes were removed by flotation and gravity, respectively. The mixture sludge/OFMSW treated was sent to the digester by a grinder pump along with the rest of the thickened sewage sludge. The biogas produced was stored and used in a cogeneration unit (190 kW_electric energy_) for heat and power production (CHP). The codigestion section operated under both mesophilic and thermophilic temperatures (this lasted for about 4 months) and under widely variable operating conditions, but almost never at full capacity (about 200 ton month^−1^).

#### 3.1.2. The Rovereto (TN) Full-Scale Food Waste Pretreatment and Anaerobic Codigestion Plant

Rovereto (TN) is a city with about 40,000 inhabitants with a production of 12.5 ton day^−1^ of OFMSW and a recycling rate of 65% [21]. The municipal wastewaters are mixed together with the wastewater produced in the industrial area and are treated in a WWTP with a design capacity of 90,000 PEs but with an effective capacity of approximately 72,000 PEs, arranged in two lines. The wastewater treatment process consists of pretreatment, primary sedimentation, alternate cycles of an activated sludge process [22], secondary sedimentation, and disinfection. The sludge produced by the primary and secondary sedimentation is thickened dynamically and sent to two mesophilic anaerobic digesters working in parallel (5000 m^3^ total working volume). The biogas produced is collected in a gas meter and converted into electricity and heat by two high-efficiency gas turbines, while the digested sludge is dewatered to approximately 23% Total Solid (TS) by centrifugation. In 2014, the Rovereto WWTP completed construction on a pretreatment section for OFMSW preparation, implementing the concept of joint processing of biowaste–sewage and anaerobic codigestion. The organic waste is pretreated with a hammer mill to remove inert and to produce a liquid flow to be pumped in the two digesters. The treatment unit is a Wackerbauer TM75, which provides the grinding/pulping of the organic substance and the separation of plastic and other residues. The operation of the equipment requires the addition of water in a ratio of about 1:1 (1 m^3^ water ton _OFMSW_^−1^). The slurry obtained has a TS content equal to 5.2% and Total Volatile Solid (TVS) of 82% of TS. This stream is treated in a hydrocyclone for the separation of sands/plastics and stored in two tanks before being fed directly to the digesters, distributing the load over 16 h per day. The residual waste flows are sent to a landfill (15%–20% wet weight). The plant was monitored in the periods before (fed with sludge only) and after the implementation of the codigestion process.

#### 3.1.3. The Camposampiero (PD) Full-Scale Food Waste Pretreatment and Anaerobic Codigestion Plant

Camposampiero WWTP has been operative since 2005: it has 35,000 PEs of wastewater treatment capacity and a wet anaerobic codigestion process working at 55 °C (biphase Linde process, 1500 m^3^ first phase and 3300 m^3^ s phase). The design data considered 16,000 ton year^−1^ of OFMSW treated together with 25,000 ton year^−1^ of manure and 7800 ton year^−1^ of thickened sludge (8% total solids). As for the pretreatment of organic waste, after a primary shredding, the material passes through an iron removal and a milling step, and then it reaches a wet-pulper. Here, the material is diluted with water or leachate from industrial organic waste. The heavy aggregates (glass, stones, etc.) are removed from the bottom of the pulper while the liquid pulp is fed to a sieve drum for the removal of light coarse materials and plastic, before reaching the first stage of the anaerobic codigestion process. The excess sludge produced by the WWTP and manure is fed directly to the thermophilic digester. In Table 1, there are data reported from the literature on AcoD process performances in order to compare them to the case studies monitored.

## 4. Results and Discussion

### 4.1. The Treviso and Rovereto (TN) Full-Scale Anaerobic Codigestion Plants

**Figure 2:**
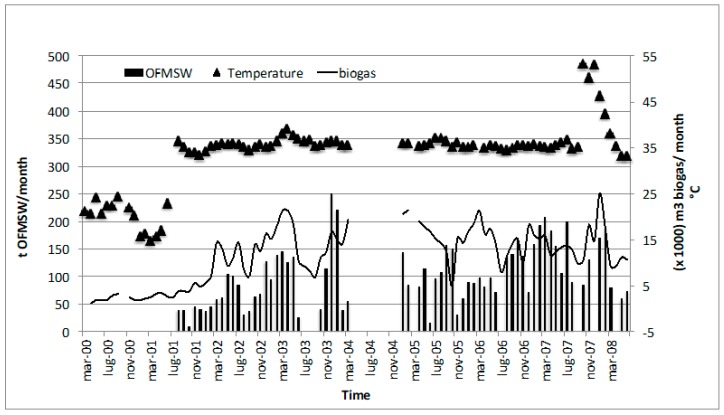
Amount of organic waste treated per month, average biogas production per month, working temperature behavior (Treviso wastewater treatment plant (WWTP)).

Over eight years of Treviso plant monitoring activity, it was possible to observe different operative process conditions: During the first year, the digester was fed with only sewage sludge and the biogas production was not sufficient to reach the mesophilic temperature (35 °C). Starting in August 2001, the OFMSW started to be fed into the anaerobic digestion process, with a consequent increase in biogas production and temperature. Mesophilic conditions were maintained during all of the years after until August 2007, when the thermophilic temperature (55 °C) was tested. The monthly biogas production trend shows a production that ranged from a minimum of 6000 m^3^ month^−1^ to a maximum of 21,000 m^3^ month^−1^ (Figure 2).

**Figure 3:**
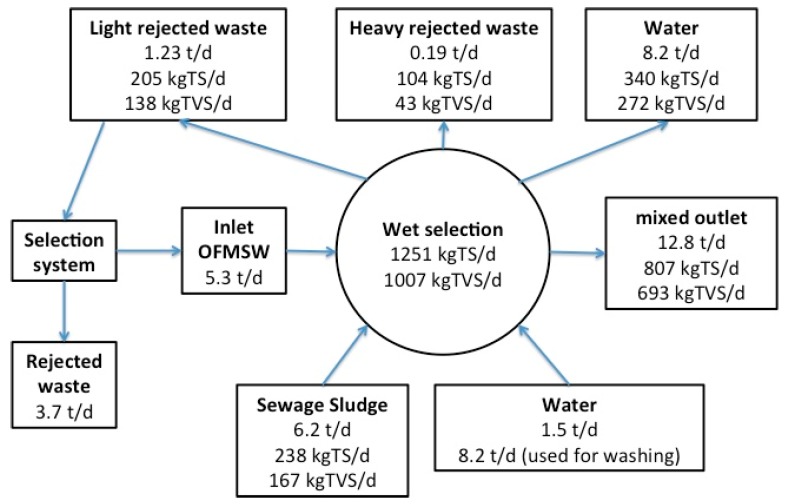
OFMSW mass balance during mechanical selection and wet refinery of Treviso plant (considering 9 ton day^−1^ of food waste treated).

The average monthly gas production was 14,097 m^3^ month^−1^, and the seven-year average specific gas production (SGP) was 0.3 m^3^ kg TVS_feed_^−1^ instead of the 0.12 typical of AD in secondary sewage sludge alone: this was double the biogas production even with a limited quantity of OFMSW. The amount of OFMSW fed into codigestion changed with time and was related to waste characteristics, to the behavior of the anaerobic process, and to the annual limit of treatment authorized at 1300 ton year^−1^. A mass balance analysis (Figure 3) of the Treviso selection system allowed for an evaluation of the selection efficiency and the content of rejected waste of the street separate collection system. The balance was done considering 9 ton day^−1^ of organic waste disposed.

**Figure 4:**
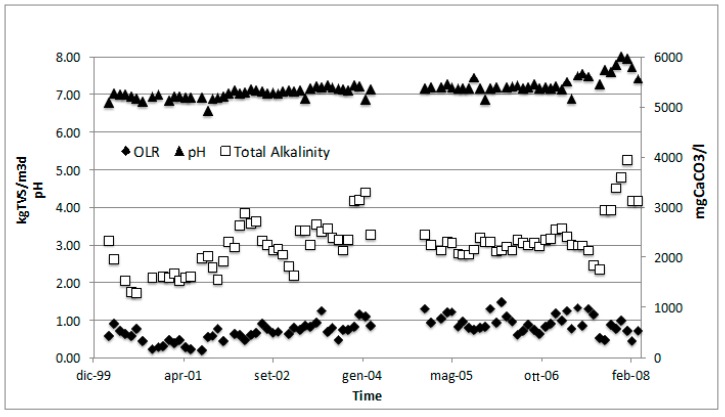
Temperature, total alkalinity, and organic loading rate behavior during eight years of anaerobic codigestion process monitoring of the Treviso anaerobic digestion (AD) process.

Forty-one percent of the 9 ton day^−1^ treated was rejected during the mechanical waste selection, and as a consequence 59% (5.3 ton day^−1^) of organic waste was sent to the wet mixer refiner. In this section, water (1.5 ton day^−1^ plus 8.2 t d^−1^ for washing) and activated sludge (6.2 ton day^−1^) were mixed together in order to decrease the solid content and to allow for heavy and light fraction separation. The mixture obtained had 65% of the inlet TS and 69% of the TVS, and in that way, 27% of TVS was in the wastewater obtained and was recirculated in the WWTP, while 8% was discharged with the heavy fraction. Depending on the season, the quality could change, with a decreased amount of rejected waste. During these seven years of mesophilic working temperatures, the process was monitored in terms of stability parameters, showing no overload conditions and increasing process stability: total alkalinity ranged between 1600 and 3300 mg CaCO_3_ L^−1^, with an average of 2328 mg CaCO_3_ L^−1^, while the pH was between 6.8 and 7.5, with an average value of 7.15 (Figure 4).

**Figure 5:**
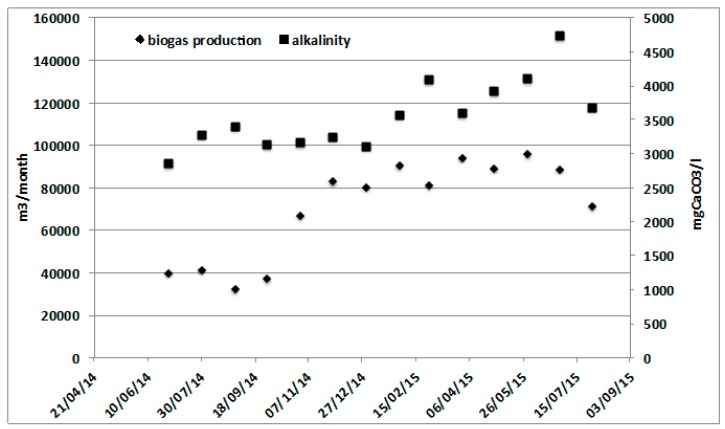
Rovereto AD biogas production changing from mono- to codigestion.

In the case of the Rovereto WWTP (Figure 5), it was possible to observe how the high biodegradability of the organic waste used as a cosubstrate ensured a doubling of biogas production and the consequent doubling of electrical energy production, driving the system toward energy autonomy, while the production of sludge to be disposed of increased only by 10%.

In Figure 5, the monthly average values of biogas production together with total alkalinity during one year of monitoring are reported. It is possible to observe that the increase in organic loading due to codigestion of the OFMSW and SS did not affect the total alkalinity, which was maintained on an almost stable level, with an average value of 3842 mg CaCO_3_ L^−1^. The energy yield passed from 4.5 to 7 MWh per day, while the energy demand for pretreatment was only 0.5 MWh per day. These figures allowed for covering the energy demand of some 35 kWh per person equivalent per year [24].

The average values of the monitored period are given in Table 2.

### 4.2. Under-The-Sink Food Waste Disposal

In terms of using the sewer system to carry the OFMSW to the WWTP, under-the-sink food disposals are of great interest, as Prof. Polanco highlighted in his keynote speech, but the use of this technique does not have particular applications in Italy. The situation is not so different in Europe: significant experiences with this transportation modality of food waste to the treatment plant have been studied. They have demonstrated the incongruity of impediments to the use of this technique through illustrations of data from some experiments carried out on a full scale. In particular, it has been noted that there are more than 110 million devices installed worldwide. Despite this fact, there are objections to the use of under-the-sink disposals concerning clogging and settling phenomena in the sewer system, the pollution of water bodies, overloading, and increased sludge production in wastewater treatment plants. A technical study emphasized that there is no problem with clogging and no need for extraordinary measures to be implemented in a sewer system as a cause of the use of under-the-sink disposals [25], there are no particular problems caused by solid loads increasing in the wastewater [26], and there is no problem with clogging, fouling, and sedimentation [27]: the sedimentation problems are related to the improper use of the equipment, as the material in the sewer system is only partially hydrolyzed and not fermented, and there are no dangerous methanogenic phenomena. Concerning objections about impacts to water bodies, it has been reported that possible problems can occur during periods of rain when the sewers are not provided with overflow systems [25]; however, these problems could be solved with the implementation of equalization tanks for overflow [27]. Concerning the impact on wastewater treatment plants, it is obvious that the increase of organic carbon and ammonia introduced with the food waste requires a larger amount of oxygen for the oxidation step, with a consequent increase of costs; however, there is an important reduction in costs for municipal solid waste disposal [25]. The anaerobic sludge stabilization performance increases significantly [26], with a consequent increase in biogas production [27]. Finally, the increased amount of rapidly biodegradable organic carbon load improves the biological nutrient removal [28,29]. Other aspects related to FWD impacts on energy consumption, water consumption, and GHG emissions have been reviewed by several authors [30,31,32,33,34,35]. The energy consumption of an FWD is variable and depends on the model, frequency, and duration of use, so it is difficult to calculate the real consumption, but in most of the studies reviewed, this value resulted in insignificance: Bolzonella et al. [36] estimated an additional energy use of 4.3 kWh and 8.5 kWh per year, meaning 0.55 € and 1.10 € per year. Water consumption depends on how much tap water is needed to produce a homogenized mixture to be flushed into the sewer: Iacovidou et al. [30] reviewed consumption ranging between 1 and 6.6 L/capita/day, which is 0.3% to 3.5% of total consumption. The GHG emissions impacts of FWDs have not been considered too much: in fact, there have been few papers on this topic, and Iacovidou et al. [26] reviewed one study in which it was estimated that the use of an FWD and anaerobic digestion generates −16.8 kg CO_2_ equiv per 100 kg of food waste compared to −1.4 kg and +74.3 kg CO_2_ equiv per 100 kg for composting and landfilling, respectively, but they took into account biogas production, carbon sequestration, and fertilizer offsets. In two life cycle assessment (LCA) studies, FWD ranked second after home composting, with 42.2 kg CO_2_ equivalent per 100 kg, and it was the best if coupled with a WWTP (compared to municipal solid waste collection followed by composting, landfilling, etc.), with 44 kg CO_2_ equivalent per 100 kg of food waste. Bernstad Saraiva et al. [31] compared two systems in the collection of food waste in households: (a) the use of food waste disposals (FWDs) in kitchen sinks and (b) the collection of food waste in paper bags for further treatment. For both cases, they considered anaerobic digestion and the use of digestate as a fertilizer. They evaluated GHG emissions from the collection and treatment of 1 ton of food waste (dry matter) and found (according to the performed assessment) lower emissions from the FWD system compared to the reference system (−990 and −770 kg CO_2_ equivalent ton^−1^ food waste dry matter, respectively). Other authors [35] have assessed different solutions for diverting food waste away from incineration toward biogasification, focusing on the feasibility of implementing FWDs in Aarhus City (Sweden) compared to transport by truck, and they concluded that the separate collection of organic waste for combined biogas and fertilizer production is the most flexible, robust, and least risky economic solution compared to FWD implementation.

In order to clarify the benefits of FWDs, Battistoni et al. [37] studied the effect before and after the implementation of an under-the-sink food waste disposal, monitoring both the sewer system and a small WWTP’s performance. The experimentation considered 95 people served and a school canteen (industrial FWD) with a 60-person equivalent capacity: the total “penetration market factor” was about 67% of the resident population. The sewage system had a retention time of 1.5 h: therefore, the time was not sufficient to trigger the fermentation process [36]. The small WWTP had a treatment capacity of 250 PE and a max flow rate of 6.9 m^3^ h^−1^, and the biological process was an intermittent controlled aeration [38,39]. The authors reported no significant solid sedimentation into pipes: therefore the hydraulic overload expected, due to the need for water for the FWD to function, did not give substantial changes, with a flow rate that was slightly leveled (maximum flow rate of 4 m^3^ h^−1^). The quality of wastewater obviously changed, and the Total Suspended Solid (TSS), Chemical Oxygen Demand (COD), and Total Nitrogen (TN) content increased 30%, 44%, and 19%, respectively, with the COD/TN ratio changing from 9.9 up to 12. In addition, the COD/TSS ratio increased from 1.4 up to 2.6. In general, they observed that the FWD technology did not overload the WWTP in any dry or wet period, and the increase in rapidly biodegradable COD could optimize the use of nitrogen-bound oxygen, which meant saving energy in the aeration system. From an economic point of view, the implementation of FWDs avoids the management costs related to source collection and transportation (from 191.400 € y^−1^ to zero) and to treatment and disposal (usually composting technology, from 47.100 € y^−1^ to 6.900 € y^−1^). Considering the capital costs of collection organization, the use of FWDs could be beneficial after 4–5 years of operation. This experience could be compared to a large-scale case study in Surhammar (Sweden): Evans et al. [40] described a “15-year” application of under-the-sink FWDs and their effects on the sewer system (plus a cost analysis). The results obtained showed that when 50% of households had an FWD installed, this meant that food waste was separated at the source and well managed. Thus, when the system reached an equilibrium with the new loading conditions, the cost effects on wastewater treatment were neutral, and with additional biogas recovery, FWDs made a positive financial contribution.

## 5. Conclusions

Smart approaches to food waste final and sustainable disposal are those able to reduce first of all the environmental impacts related to waste management: in this context, the application of an under-the-sink food waste disposal avoids transportation pollution and management costs. The second smart aspect is energy recovery through the anaerobic codigestion of food waste and sewage sludge, together with a reduction in CO_2_ emissions (if compared to composting technologies). Considering the long-term successful applications of anaerobic codigestion processes within the Italian territory, it is possible to conclude that from technological and environmental points of view, there are no further impediments for new applications of these approaches. Some important evaluations must be carried out related to the application of an effective separate collection, the choice of appropriate food waste pretreatments, and an economical evaluation of the whole process, considering incentives for biogas exploitation and the revenue achievable from these processes from other new products. Food waste is a renewable resource, and through its valorization, it is possible to transform wastewater treatment plants into small biorefineries able to manage waste systems in a sustainable way, with interesting economic revenue for citizens.

## Figures and Tables

**Table 1:** Camposampiero anaerobic digestion process performance [23]. OFMSW: organic fraction of municipal solid waste.

Parameter	AcoD of Sludge, Manure, and OFMSW at Thermophilic Range
Hydraulic Retention Time (HRT), day	22
Organic Loading Rate (OLR), kg TVS m^3^ day^−1^	3.52
Specific Gas Production (SGP), Nm^3^ kg TVS^−1^	0.67
Gas Production Rate (GPR), Nm^3^ m^−3^ day^−1^	1.46
CH_4_, %	58–60

**Table 2:** Treviso (seven years) and Rovereto (one year, [24]) anaerobic digestion process performance monitoring. TS: total solids; TVS: total volatile solids.

Parameters	Unit	Treviso	Rovereto
Temperature	°C	35.7	35–37
pH		7.15	7.42
Total alkalinity	Mg CaCO_3_ L^−1^	2328	3842
Total solids	g L^−1^	30.1	23.98
Total volatile solids	g L^−1^	15.8	16.34
TS/TVS	%	52.4	68
Biogas production	m^3^ month^−1^	14,097	86,070
Gas production rate (GPR)	m^3^ m^3^_reactor_ day^−1^	0.21	0.43
Specific gas production (SGP)	m^3^ kg TVS^−1^	0.3	0.5
Hydraulic retention time (HRT)	day	25–30	30–40
Organic loading rate (OLR)	kg TVS m^3^_reactor_ d^−1^	0.87	1.38
OFMSW collected	ton month^−1^	107.7	

## Abbreviations

AcoD	anaerobic codigestion
AD	anaerobic digestion
BNR	biological nutrient removal
COD	chemical oxygen demand
FWD	food waste disposer
GHG	greenhouse gas
GPR	gas production rate
HRT	Hydraulic retention time
LCA	Life cycle assessment
OFMSW	organic fraction of municipal solid waste
OLR	Organic loading rate
PE	people equivalent
PHA	polyhydroxyalcanoate
rbCOD	rapidly available chemical oxygen demand
SBR	sequencing batch reactor
SGP	specific gas production
SS	sewage sludge
TN	total nitrogen
TS	total solid
TSS	total suspended solid
TVS	total volatile solid
VFA	volatile fatty acid
WWTP	wastewater treatment plant
